# Behavioural and environmental risk factors associated with snake–human conflict: A scoping systematic review

**DOI:** 10.1371/journal.pntd.0014505

**Published:** 2026-08-06

**Authors:** Chandrashekhar Janakiram, Arjun Satheesh Chandran Nair, Amrutha V. Nair, Ajay Pious, Geetha Gireesh Gyra, Anju James, Srikanta Kanungo, Rahul K. Gajbhiye, Nirmalya Mukherjee, Omesh Kumar Bharti, Surajit Giri, Sunil Panigrahi, Jaideep C. Menon

**Affiliations:** 1 Director, Amrita JBI Centre for Evidence Synthesis and Implementation, India Amrita Vishwa Vidyapeetham, Kochi, India; 2 Project Research Scientist, ICMR – D-SERPENT project, Dept of Adult Cardiology and Public health, Amrita Institute of Medical Sciences, Amrita Vishwa Vidyapeetham, Kochi, India; 3 Senior Project Assistant. ICMR – D-SERPENT project, Dept of Adult Cardiology and Public health, Amrita Institute of Medical Sciences, Amrita Vishwa Vidyapeetham, Kochi, India; 4 Resident Department of Public Health Dentistry, Amrita School of Dentistry- Amrita Vishwa Vidyapeetham, Kochi, India; 5 Additional Reader, Department of Public Health Dentistry, Amrita School of Dentistry- Amrita Vishwa Vidyapeetham, Kochi, India; 6 Scientist D, ICMR- Regional Medical Research Centre, Bhubaneswar, Odisha, India; 7 Scientist E, ICMR-National Institute for Research in Reproductive and Child Health, Mumbai Nodal Officer, Model Rural Health Research Unit, Vani District Nasik, Maharashtra, India; 8 Chief Executive, CPHR-MANT, Kolkata, West Bengal, India; 9 Dy. Director and Epidemiologist Himachal Pradesh Health Department of Health and Family Welfare, Himachal Pradesh, India; 10 Consultant Anaesthesiologist, National Health Mission Govt of Assam, Guwahati, India; 11 Assistant Professor Department of Community Medicine, AII India Institute of Medical Sciences Deoghar Jharkhand, Deoghar, India; 12 Dept of Adult Cardiology and Public health, Amrita Institute of Medical Sciences, Amrita Vishwa Vidyapeetham, Kochi, India; National University of Singapore, SINGAPORE

## Abstract

**Background:**

Snakebite envenoming remains a major yet neglected public health problem, disproportionately affecting rural and agrarian populations in low- and middle-income countries. While clinical management and antivenom availability have received increasing attention, far less emphasis has been placed on synthesising the upstream behavioural, environmental, occupational, and socioeconomic circumstances that shape snake–human conflict and lead to snakebite. Existing evidence on these circumstances is fragmented, heterogeneous, and context-specific, limiting its utility for informing prevention strategies. A comprehensive mapping of reported circumstances associated with snake–human conflict is therefore essential to support primary prevention, policy development, and future analytical research.

**Objectives:**

This scoping review aimed to map and synthesise the available evidence on behavioural and environmental circumstances associated with snake–human conflict among populations living in snake-endemic settings across diverse geographic and socioecological contexts. Specifically, the review sought to (i) describe the range of reported circumstances under which snake–human conflict occurs, (ii) characterise the populations and contexts in which these encounters take place, and (iii) identify gaps in the existing evidence base.

**Methods:**

A scoping review was conducted in accordance with the Joanna Briggs Institute methodology and reported following the PRISMA Extension for Scoping Reviews (PRISMA-ScR). A comprehensive search of electronic databases (including PubMed, Embase, Scopus, Web of Science, CINAHL, and Global Index Medicus) and grey literature sources was under taken without date restrictions. Observational studies, descriptive epidemiological studies, community surveys, surveillance analyses, mixed-methods studies, and qualitative research reporting behavioural, environmental, occupational, or socioeconomic circumstances related to snakebite were eligible for inclusion. Two reviewers independently screened records, assessed full texts, and charted data using a standardised extraction framework. Extracted data were synthesised using descriptive numerical summaries and narrative thematic mapping across four domains: environmental, occupational, behavioural, and socioeconomic determinants. No critical appraisal or effect estimation was undertaken, consistent with scoping review methodology.

**Results:**

A total of 142 studies met the inclusion criteria, representing evidence from Asia, Africa, the Americas, Australia, and Europe, with the majority originating from South Asia. The synthesis included **47892** reported snakebite circumstances, 15931 time-of-bite records, 85593 anatomical site records, and 1,16,759 snake identification records. Agricultural or farm-related activities (59.1%), working (9.1%), and walking (8.4%) were the most frequently reported circumstances associated with snakebite. Day-time bites were slightly more common than night-time bites (54.6%), with a peak between 12 PM and 6 PM. The lower limb accounted for 79% of reported bite sites. Reported circumstances clustered into four interconnected domains: environmental factors (seasonality, climate, housing, land use), occupational exposures (agriculture, plantation work, forestry, manual labour), behavioural practices (barefoot walking, night-time activity, sleeping on the floor, snake handling), and socioeconomic vulnerability (poverty, limited education, rurality, constrained healthcare access).

**Conclusions:**

This scoping review demonstrates that snake–human conflict and snakebite occur within predictable socioecological contexts shaped by interacting environmental, occupational, behavioural, and socioeconomic determinants. The findings highlight opportunities for prevention through integrated, context-specific strategies addressing environmental management, occupational safety, behaviour change, and structural vulnerability. While the review does not estimate risk or causality, it provides a consolidated evidence map to inform policy, programme design, and future analytical research aimed at reducing the global burden of snakebite envenoming.

## Author summary

Snakebite envenoming continues to impose a substantial public health burden, particularly among rural and agrarian populations in low- and middle-income countries. This scoping review systematically mapped and synthesised the behavioural, environmental, occupational, and socioeconomic circumstances associated with snake–human conflict while identifying key evidence gaps in the existing literature. The review was conducted in accordance with the Joanna Briggs Institute methodology and reported following the PRISMA-ScR guidelines, incorporating evidence from 142 studies spanning Asia, Africa, the Americas, Australia, and Europe. Agricultural activities emerged as the most frequently reported circumstances associated with snakebite, with bites occurring predominantly during daytime and most commonly affecting the lower extremities. Consistent determinants of snakebite included seasonal and climatic factors, agricultural and manual occupations, barefoot walking, nocturnal activities, sleeping on the floor, intentional snake handling, poverty, rural residence, and limited access to healthcare services. Overall, the evidence demonstrates that snake–human conflict occurs within predictable socioecological settings shaped by the complex interaction of environmental exposures, occupational practices, and social vulnerabilities. These findings provide a comprehensive synthesis of the circumstances surrounding snakebite occurrence and offer an evidence base to inform context-specific prevention strategies, public health policy, and future analytical and implementation research.

## Introduction

Snakebite envenoming (SBE) is a major public health issue that disproportionately affects low- and middle-income countries (LMICs), particularly rural and agricultural communities in sub-Saharan Africa, South and Southeast Asia, and Latin America.[1] Despite its official recognition as a neglected tropical disease (NTD) by the World Health Organization (WHO), snakebite has continued to receive limited attention in prevention strategies, policy prioritisation, and research funding. The global burden of snakebite is substantial, with WHO estimates suggesting that approximately 5.4 million snakebites occur annually, resulting in up to 2.7 million envenoming’s and between 81,000 and 1,38,000 deaths worldwide.[1] In addition to mortality, hundreds of thousands of survivors experience long-term disabilities, including amputations, chronic pain, and psychological trauma, which have profound implications for individuals, families, and health systems.[2]

India accounts for nearly half of global snakebite mortality, with an estimated 58,000 deaths annually.[3] Most snakebite victims in India are drawn from rural and agrarian communities, including farmers, agricultural labourers, and forest workers, who frequently lack timely access to appropriate medical care.[1] In a country where agriculture employs more than 50% of the workforce and where large populations reside in snake-endemic regions, understanding the circumstances under which snake–human conflict occurs is essential for developing effective and contextually appropriate prevention strategies. Beyond clinical outcomes, snakebite-related morbidity and mortality impose significant social and economic burdens on households and health systems, exacerbating cycles of poverty and vulnerability.[4]

Although clinical management and antivenom therapy have historically dominated snakebite research, comparatively less attention has been directed toward upstream determinants of risk. In particular, the behavioural and environmental circumstances that shape snake–human interactions have not been comprehensively mapped. Existing studies point to a complex interplay of environmental, occupational, behavioural, and socioeconomic factors that influence when, where, and how snake–human conflict occurs. However, this evidence remains fragmented, context-specific, and unevenly distributed across regions, making it difficult to derive a consolidated understanding of the circumstances associated with snake–human conflict, especially in high-burden settings.

Environmental conditions play a central role in shaping snakebite risk and snake–human encounters. [5] Seasonal patterns, particularly during monsoon and post-monsoon periods, have been consistently associated with increased snakebite incidence. Flooding displaces both human populations and snakes, increasing the probability of contact. [6] In India and Southeast Asia, snake activity peaks during rainy seasons when prey availability increases and human agricultural activity intensifies.[7] For example, evidence from Tamil Nadu, India has shown that more than 70% of snakebite cases occurred between June and September.[8] Land-use changes, including deforestation and habitat degradation, further contribute to snake displacement into human settlements, intensifying opportunities for snake–human conflict.[5]

Geographical and housing-related environmental features, including proximity to forests, rice paddies, water bodies, and poorly maintained dwellings have been identified as high-risk settings for snake encounters. Rural housing characterised by mud floors, open ventilation, and thatched roofing facilitates snake entry into living and sleeping spaces. For instance, a case–control study from Bangladesh demonstrated that sleeping on the floor increased the risk of snakebite by more than fourfold.[9] Importantly, these environmental circumstances vary across ecological zones, climatic conditions, and snake species distributions, underscoring the need to systematically map context-specific patterns rather than assume uniform risk profiles.

Occupational circumstances represent another critical dimension of snake–human conflict. [10] Agricultural workers, plantation labourers, herders, forestry workers, and construction labourers are routinely exposed to snake habitats as part of their daily livelihoods. [11] These occupational groups often work barefoot or with minimal protective equipment, increasing vulnerability to snakebite. Evidence from Nepal indicates that farmers and manual labourers accounted for over 70% of reported snakebite victims, with similar occupational patterns reported in Sri Lanka, Nigeria [12], and Brazil [13]. Such findings highlight the importance of understanding how livelihood activities intersect with environmental contexts to shape exposure.

Behavioural circumstances further compound risk and act as immediate triggers for snake–human encounters. Practices such as walking barefoot, sleeping outdoors or on the floor, working without protective gloves, and moving outdoors at night without adequate lighting are frequently reported across snakebite studies.[14] In rural India, children are particularly vulnerable due to outdoor play and unsupervised movement during early morning and evening hours. [15,16] Limited awareness of snake behaviour, first aid practices, and appropriate treatment-seeking further exacerbates morbidity and mortality following snakebite events.[17]

Socioeconomic conditions underpin and reinforce environmental, occupational, and behavioural circumstances. Poverty, low literacy, and limited access to healthcare shape both exposure and outcomes of snake–human conflict. In resource-constrained settings, delays in reaching health facilities, reliance on traditional healers, and inability to afford antivenom treatment can be fatal.[18] Evidence from multicentric studies in India indicates significantly higher snakebite-related mortality among low-income households, even after accounting for geographic and occupational factors.[19] Cultural beliefs and health system constraints further influence care-seeking behaviours, often delaying lifesaving treatment.

Despite the recognised burden of snakebite and the established importance of social and environmental determinants in other public health domains, the behavioural and environmental circumstances associated with snake–human conflict have not been comprehensively mapped. Existing studies vary widely in design, populations, definitions, and outcomes, limiting the ability to draw generalisable conclusions. The WHO strategy for snakebite envenoming has called for a 50% reduction in mortality and disability by 2030, a target that cannot be achieved through clinical management alone. Primary prevention efforts require a nuanced understanding of where, when, and under what circumstances snake–human conflict occurs.

A scoping review is particularly appropriate to map the breadth of existing evidence on behavioural and environmental circumstances related to snake–human conflict. By systematically identifying, categorising, and synthesising the available literature, this review aims to provide a comprehensive overview of reported circumstances, populations, and contexts in which snake- human conflict occurs. This approach will help identify evidence gaps, highlight regional variations, and inform future research priorities, targeted prevention strategies and policy development. Ultimately, the review seeks to establish a typology of behavioural and environmental circumstances associated with snake–human conflict and support context-sensitive public health responses across endemic settings. While the review did not estimate risk or causality, it mapped commonly reported circumstances associated with snake human conflict to inform future risk-factor analyses.

### Review questions

What behavioural and environmental circumstances related to snake–human conflict has been reported among human populations living in snake-endemic settings across different geographic and socioecological contexts?

## Objectives

To map the range of behavioural and environmental circumstances associated with snake-human conflict reported in the literature.

To describe the contexts and populations in which snake-human conflict occurs.

To identify key gaps in evidence and areas requiring further research.

## Methods

### Study design

A scoping review approach was selected to systematically map the breadth, characteristics, and contexts of existing evidence on behavioural and environmental circumstances associated with snake–human conflict, rather than to assess causality or intervention effectiveness. This review was conducted as a scoping review in accordance with JBI methodology.[20] While the review did not aim to estimate pooled effect sizes or establish causality, it incorporated structured mapping of behavioural and environmental circumstances associated with increased snakebite risk, drawing on evidence from observational studies. This approach allowed identification of recurrent risk patterns and contexts while maintaining the exploratory scope of a scoping review.

### Types of sources

Consistent with JBI scoping review guidance, a broad range of evidence sources was included. These comprised observational studies (cross-sectional, cohort, and case–control), descriptive epidemiological studies, community-based surveys, surveillance data analyses, mixed-methods studies, and qualitative research reporting contextual aspects of snake–human conflict. Case reports and case series were included when they provided relevant contextual information. Editorials, commentaries, and opinion pieces were excluded but were reviewed for citation tracking.

### Search strategy

A comprehensive search strategy was developed following JBI’s three-step approach. An initial limited search of MEDLINE (via Ovid) and CINAHL was undertaken to identify relevant keywords and index terms. This was followed by a full search across multiple electronic databases, including PubMed, Embase, Scopus, Web of Science, CINAHL, and Global Index Medicus. Grey literature was identified through Google Scholar, institutional repositories, and reports from national and international health agencies. Reference lists of included sources were screened for additional evidence. No date restrictions were applied. Only studies published in English were included.

### Study selection

All identified records were imported into reference management software JBI SUMARI, and duplicate records were removed using the zotero. Titles and abstracts were independently screened by two reviewers against the eligibility criteria. Full-text articles of potentially relevant sources were retrieved and assessed in detail. Disagreements were resolved through discussion, with consultation of a third reviewer where necessary. The study selection process was documented using a PRISMA-ScR flow diagram [21].

### Data charting

Data were charted using a customized data extraction form developed in accordance with JBI guidance and refined iteratively as familiarity with the literature increased. Two reviewers independently charted data, and discrepancies were resolved by consensus. Extracted data included study characteristics (author, year, country, design, duration, and sample size), population characteristics, and detailed descriptions of behavioural and environmental circumstances associated with snakebite. Outcome data included circumstances of bite, time of bite, anatomical site, and snake species involved. Discrepancies were resolved by consensus.

### Data synthesis and presentation

Findings were synthesised using descriptive numerical summaries and narrative thematic mapping, consistent with JBI scoping review methodology. Quantitative data were summarised using frequencies and proportions to describe distributions of circumstances of snakebite, timing of bites, anatomical sites, and snake types. Qualitative and contextual data were analysed thematically and organised into four overarching domains: environmental, occupational, behavioural, and socioeconomic determinants. Results were presented using summary tables, a risk factor classification matrix, an evidence-to-risk mapping matrix aligned with the PCC framework, and conceptual figures, including a Snake–Human Conflict Pathways Framework and a causal loop diagram. Descriptive numerical summaries were generated to map the distribution of reported snakebite circumstances, temporal patterns, anatomical sites of bites, and snake species involved. No exposed–unexposed comparisons, effect estimates, or pooled measures of association were calculated. Consistent with JBI scoping review methodology, the numerical summaries describe the extent and nature of the evidence base and should not be interpreted as measures of risk or causality.

## Results

The study synthesised data from 214708 recorded snake-bite incidents, capturing the circumstances, timing, anatomical site, and snake species involved. Results are presented as descriptive summaries of reported circumstances and contexts of snakebite and do not represent comparative measures of risk.

### Study selection

**Fig 1:**
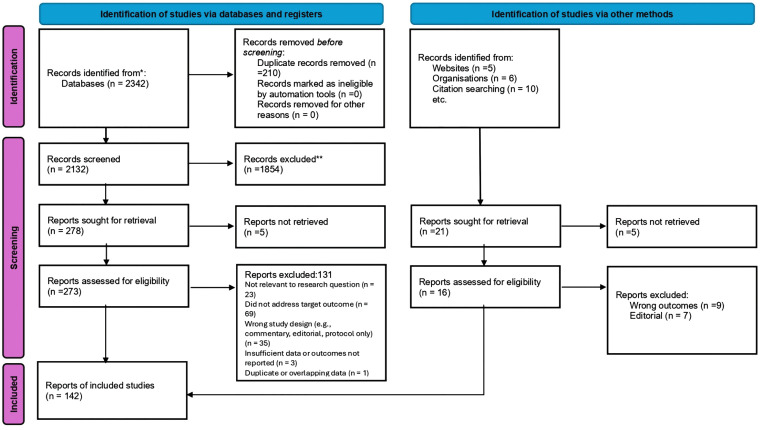
The study selection Process.

**Fig 2:**
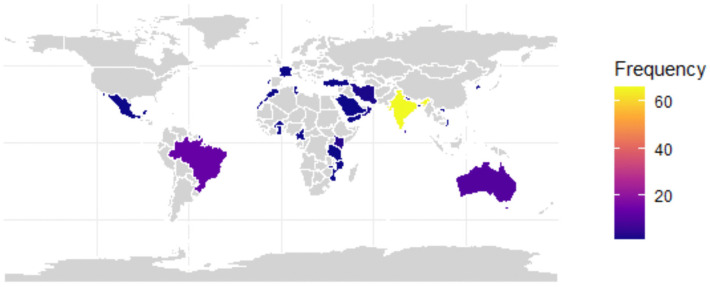
The global distribution of included studies. Fig 2. The map was created using R (ggplot2 package) with country boundary data obtained from Natural Earth (https://www.naturalearthdata.com/), accessed via the rnaturalearth package. Natural Earth data are in the public domain and are free for use without restriction (https://www.naturalearthdata.com/about/terms-of-use/). The visualization was generated using original study data.

The database search identified 2,342 records, with an additional 21 records identified through other sources, including organisational websites and citation searching. After removal of 210 duplicate records, 2,132 records were screened at the title and abstract level, of which 1,854 were excluded for not meeting the predefined inclusion criteria. A total of 278 reports were sought for full-text retrieval, and five reports could not be retrieved. Consequently, 273 full-text reports were assessed for eligibility. Following full-text assessment, 142 reports were excluded for specific reasons, including failure to address the target outcome, ineligible study designs (such as editorials, commentaries, or protocols), lack of relevance to the research question, insufficient outcome data, or duplicate or overlapping data. A further 3 reports were excluded due to incorrect outcomes or editorial nature. Ultimately, 142 studies met the eligibility criteria and were included in the final review Fig 1 and 2.

### Characteristics of included studies

The search identified a total of 142 studies that met the inclusion criteria for this review. These studies encompassed a wide geographic spread across Asia, Africa, the Americas, and Australia, although the majority (over 60%) originated from India, reflecting the country’s substantial global burden of snakebite. Study designs varied, comprising cross-sectional surveys, retrospective and prospective observational studies, cohort studies, descriptive epidemiological analyses, nested case–control studies, community-based surveys, and a limited number of case series and case reports. All included studies provided relevant data on environmental, occupational, behavioural, or socioeconomic determinants associated with snakebite incidence. A detailed summary of the characteristics of the included studies is presented in the compiled table of study characteristics.

The earliest included study dated back to 1987, whereas the most recent were published in 2025, demonstrating a multi-decade research landscape. Sample sizes ranged widely, from single-patient case reports to national surveillance datasets with more than 70,000 recorded cases. Several large-scale community surveys provided population-level estimates, while numerous hospital-based retrospective studies contributed clinical and epidemiologic insights. Study durations ranged from short periods of several months to extended data collection spanning more than a decade.

A substantial proportion of included studies evaluated the contextual or ecological determinants of snakebite, including associations with agricultural work, seasonal climatic variation, land-use patterns, housing, and occupational exposures. Behavioural factors such as barefoot walking, sleeping on the floor, poor lighting, and intentional snake handling were frequently reported across settings. Table 1

**Table 1:** Characteristics of Studies Included in the review.

Serial No.	Study ID	Country	Design	Study duration	Sample Size
1	MCNALLY, SL, 1987	South Africa	Retrospective study	January 1978 to December 1982.	251
2	Ribeiro,1995	Brazil	Descriptive study	1988	531
3	Sutherland,1995	Australia	Retrospective case review	1992-1994	12
4	Saborío, P, 1998	Costa Rica	Retrospective study	1985 - 1995	80
5	Bhardwaj, A 1998	India	descriptive case series	24 months	243
6	Bakshi S.A., 1999	India	Descriptve Study	July 1994 to October 1994	138
7	Chippaux, 2002	Africa	Retrospective-Prospective	1985 - 1997	403800
8	Kularatne,2002	Sri Lanka	Prospective Study	1996 - 1998	210
9	Bawaskar H.S., 2002	India	Retrospective/Descriptive Study	January 1998 to January 2001	91
10	da Silva,2003	Brazil	Retrospective - Prospective Study	1993–1995	92
11	Bawaskar,2004	India	Observational/Case Study	2001–2003	30
12	Sharma, 2004	Nepal	Retrospective Study	2001 december	10,550
13	Moreno, 2005	Brazil	Prospective observational/case series study	January to December 2002	144
14	Ozay G, 2005	Turkey	Retrospective Observational Review	May 1997 and May 2002	77
15	Punde, D.P, 2005	India	Retrospective analysis	1992 - 2001	633
16	Sharma N,2005	India	Retrospective case note analysis	January 1997 to December 2001	142
17	Sloan, DJ, 2007	South Africa	Retrospective Review	Incidence review: 1 January 2000 to 31 December 2005. Interview period: April to December 2005.	744
18	Suchithra,2008	India	Prospective Cohort Study	May 2005 - December 2006	635
19	Robert A. Harrison, 2009	Global focus (primarily sub-Saharan Africa, South Asia, Southeast Asia, Latin America)	Literature review and global burden discussion	upto 2009	no primary data collection
20	Al-Lawati, 2009	Oman	Retrospective Study	May 2006 to August 2008	65
21	Churchman A; 2010	Australia	Prospective cohort observational study	January 2002 to June 2010	81
22	Rahman,2010	Bangladesh	Cross-Sectional Survey	February to June 2009	18,857
23	2011 Karlo, Robert;	Croatia	Retrospective study	1999–2009	93
24	Chattopadhyay,2011	India	Prospective Study	January 2006 to December 2008	86
25	Mohapatra,2011	India	Representative study	2001-2003	1,22,848
26	Monteiro,2011	India	Prospective Research Study	August-2003 to November-2005	4
27	Isbister GK, 2012	Australia	Prospective, observational study	2004–2011	1089
28	Isbister GK, 2012	Australia	Prospective multicenter cohort study	October 2004 – June 2011	60
29	Haidar, 2012	Yemen	Retrospective Study	March 1997 – April 2001	186
30	Cassiano de Brito, 2012	Brazil	Cross Sectional	January 2007 - December 2011	2106
31	Monteiro,2012	India	Prospective Study	August 2003 to November 2005.	95
32	Vaiyapuri, S 2013	India	Community survey	10 years (prior to the survey)+ household surveys in November and December 2010.	28,494
33	Rao,2013	India	Retrospective Study	5 years	60
34	Halesha, 2013	India	Retrospective/Descriptive Study	January 2010 to December 2011	180
35	de Almeida Graciano, 2013	Brazil	Quantitative, exploratory, retrospective documentary study	2006–2009	220
36	Rajesh Kumar,2014	India	Retrospective Study	2010–2012	87
37	Majumder,2014	India	Retrospective Study	January 2009 to October 2010	4,871
38	Raina,2014	India	Retrospective-Descriptive Study	July 2010 to June 2012	200
39	Kularatne, 2014	Sri Lanka	Prospective-Cohort Study	January 2010 - december 2010	1,018
40	Mitra S, 2015	India	Retrospective-Descriptive Study	January 2007 to December 2012	356
41	Mohankumar,2015	India	Prospective/Retrospective Study	January 2013 to December 2013	164
42	Thapar,2015	India	Retrospective study	January 01, 2007 to December 31, 2011	198
43	Al Hatali,2015	Oman	Case Report	case report	1
44	Feitosa, 2015	Brazil	Nested Case-Control Study	January 1, 2007 to December 31, 2012	9,191
45	Gupt,2015	India	cross sectional study	2008–2012	497
46	Spyres,2016	USA	Prospective Case Series	January 1, 2014, and November 5, 2015	25
47	Wood, D, 2016	South Africa	Retrospective case series	September 2008 to December 2013.	879
48	Pandey P.C, 2016	India	Descriptive(Retrospective) Study	Not mentioned	113
49	Prakash A.K.S, 2016	India	Cross-sectional, descriptive study	January 2012 to November 2014	46
50	Chandrakumar,2016	India	Cross-Sectional study	February 2012- August 2014	91
51	Digra,2016	India	Retrospective study	November 2007 to October 2011	138
52	Manigandan, 2016	India	Retrospective study	January 1, 2014, to December 31, 2015	108
53	Mukhopadhyay P, 2016	India	Prospective study	April 2010 to July 2011	155
54	Farooqui,2016	India	Retrospective Study	January 2004 to December 2014	54
55	Claudet, I, 2016	France	Retrospective-Cohort Study	24 April 2001 to 16 July 2014	83
56	Pulimaddi, R, 2017	India	Prospective, observational study	November 2013 to July 2015	100
57	Welton, 2017	Australia	Retrospective coronial database analysis	3 years	35
58	Johnston, 2017	Australia	Prospective Study	July 2005 to June 2015	1,548
59	Ruha, 2017	USA	Descriptive Report	January 1, 2013 and December 31, 2015	450
60	Sarkhel,2017	India	Retrospective Study	January 2012 to December 2016	1,633
61	El Zahran,2018	Lebanon	Retrospective chart review	Between January 1, 2000, and September 30, 2014	24
62	Pandey, DP, 2018	Nepal	Mixed Methods	November 2011 through December 2014.	56
63	El Hattimy,2018	Morocco	Retrospective Study	1999–2013	2,053
64	Ghosh,2018	India	Retrospective Descriptive Epidemiology Study	January 2012 to December 2016	222
65	Dandona, R, 2018	India	prospective cohort study	January 2012 to March 2014	6,27,658
66	Kumar,2018	India	Prospective Observational study	January 2012 to December 2016	1,500
67	Kassiri, 2019	Iran	Cross-Sectional Descriptive-Analytical Study	2013 and 2017	102
68	Sahu, G; 2019	India	cross sectional - prospective study	Community survey: Oct 2014 – Nov 2015 (13 months) • Hospital study: Jul 2014 – Mar 2016	35163
69	Gajbhiye,2019	India	Retrospective Study	January 2014 to December 2014	145
70	Majumdar, 2019	India	Observational - Cross sectional Study	February 2011 - January 2012	120
71	Mishra, 2019	India	Descriptive Study	2019	89
72	Gantait, 2019	India	Prospective Observational study	January 2012 – December 2015	1220
73	Tchoffo,2019	Central Africa	Observational study	July 2015 to June 2016	516
74	Lath V, 2019	India	Retrospective Study	2010 – 2014	454
75	da Costa,2019	Brazil	Retrospective Study	2007–2016	3,909
76	Yakubu, 2019	Ghana	Descriptive cross-sectional review of medical records	3 years	86
77	Musah,2019	Africa	Cross-Sectional Study	Community Survey: December 2008 – May 2009. Retrospective Data: 10 years (1999–2008)	1,024
78	Senek,2019	South Korea	Cross-sectional, descriptive, population-based study	1 January 2011 – 31 December 2016	1335
79	Kautilya, 2019	India	Retrospective Study	January 2013 to December 2013	184 households
80	Mendez-Dominguez,2019	Mexico	Case Report	not provided since its a single case report	1
81	Panwar, 2019	India	Retrospective Study	January 2013 and October 2018	102
82	Alcoba, 2020	Cameroon	Cross-Sectional Study	October to December 2016 (data from July 2015 – June 2016)	9,924
83	Annal, 2020	India	Case Report	2020	1
84	Kharat,2020	India	Prospective Observational Study	July 2017 to June 2018	245
85	Sinha S, 2020	India	Retrospective Study	June 2015 to July 2019	108
86	Pathak H.S, 2020	India	Retrospective-Descriptive Study	April 2013 to March 2014	160
87	Jayawardana,2020	Sri Lanka	Cross-Sectional Study	12 months	8707
88	Feitosa,2020	Brazil	Ecological study	2007–2015	7,764
89	Bisneto, PF, 2020	Brazil	Retrospective study	January 2010 to December 2015	70,816
90	Ferreira,2020	Brazil	Retrospective-Cross Sectional study	January 2007 to December 2018	2655
91	Vishwakarma, 2020	India	Retrospective Study	2016-2020	58
92	Gutierrez, 2021	Costa Rica, Sri Lanka, Nigeria	Narrative review and comparative analysis of national snakebite contexts	multiple years	National estimates
93	Wasko, 2021	USA	Retrospective-Descriptive Study	1800 – 2015	848
94	Carvalho,2021	Portugal	Case Report	Case report	1
95	Dalal,2021	India	Descriptive Study	July 2014 to June 2016	125
96	Suryanarayana,2021	India	Retrospective Cohort Study	June 2009 to July 2015	308
97	Melit,2021	India	Retrospective Study	January 2010 to January 2015	3229
98	Rubeshkumar P, 2021	India	Secondary Data Analysis	2009–2018	Covers all reported snakebite cases and deaths in India via the National Health Profile (NHP)
99	Sathish K, 2021	India	autopsy-based prospective descriptive study	January 2017 to December 2018	38
100	Greene,2021	USA	Descriptive Report	January 1, 2015, - December 31, 2019	14
101	GI ooms, 2021	Kenya	Cross-Sectional	2020	382
102	Nduwayezu, 2021	Africa	Retrospective Study/Cross Sectional Survey	2017 & 2018	363
103	Witharana,2021	Sri Lanka	Prospective Observational study	November 2013 to October 2015	83
104	Tiemensma,2021	Australia	Case Report	Single case report	1
105	Chakroun-Walha, 2021	Tunisia	Retrospective Observational Study	January 1, 2014 – December 31, 2019	109
106	Johnston,2022	Australia	Prospective Observational study	January 2002 -August 2020	13
107	Magee,2022	Australia	Case Report	case report	24
108	Jestrzemski, 2022	Republic of Cyprus	Retrospective study	2013–2019	288
109	Schurer,2022	Africa	Qualitative Study	2013-2018	105
110	SFV Magalhães, 2022	Brazil	Analytical Epidemiological Study	2010–2015	62,591
111	Larson, PS; 2022	Kenya	Mixed Method Study	survey - about snakebites in the 5 years previous to the visit.	396
112	Harith,2022	Africa	Cross-Sectional	1 year - 2019	7544
113	sirur,2022	India	Case Series	Not explicitly stated	3
114	Oliveira, 2023	Brazil	Clinical Review	2007–2021	42,257
115	Francis, 2023	Tanzania	Mixed-methods study: Interviews with snakebite victims/guardians and health professionals, and analysis of retrospective patient case records from a clinic.	2007–2019	101
116	ralph,2023	India	Case Report	2020	1
117	Mukhopadhyay,2023	India	Descriptive,Observational,Cross-sectional	September,2017 to August, 2018	49
118	OBryan,2023	Mozambique	Descriptive analysis	2021	70,947
119	Kakati,2023	India	Retrospective Study	April 2018 to August 2022	1,011
120	Giri,2023	India	Case Report	NA	1
121	Mahapatra,2023	India	Cross Sectional Study	July 2019-June 2020	201
122	Pandey, 2023	Nepal	Prospective cohort study	May 2017 to December 2017	476
123	Hsu,2023	Taiwan	Retrospective epidemiological study	January 2002 to December 2014	12,542
124	Mandal, 2023	India	Retrospective study	May 01, 2014, and May 01, 2019	80
125	Stephen,2024	India	Prospective-Observational Study	June 2021, to May 2022	145
126	Muniyapillai,2024	India	Retrospective medical record review	January 1, 2021, to December 31, 2023	202
127	Avunoori Pushpalatha, 2024	India	Prospective Observational study	1 year	68
128	Kinda, 2024	Burkina Faso	Cross-sectional study using secondary hospital data	5 years	1118
129	Jain,2024	India	Cross- Sectional Study	March 2020 to November 2020	30
130	kumar,2024	India	Case Series	NA	3
131	Ralph,2024	India	Case Report	2023	2
132	Hakizimana,2024	Africa	Cross-sectional – Retrospective study	year 2020	3,90,546
133	Alfaifi, 2024	Saudi Arabia	Retrospective study	January 2000 – December 2021	69
134	Tiany,2024	Kenya	Cross-Sectional study	December2019 to March2020	3,610
135	Le,2025	Vietnam	Retrospective Study	2008–2020	5805
136	Patra, 2025	India	Cross-Sectional	December 2020 to December 2021	2301
137	Kabeya,2025	Africa	Descriptive, Retrospective, Observational study	1 September 2019 to 31 August 2022	155
138	Sasidharan,2025	India	Retrospective-Prospective Observational Study	July 2021 to July 2022	101
139	Shaban,2025	Iran	Descriptive-Analytical Retrospective Study	2015–2022	34
140	Naik,2025	Africa	Retrospective, cross-sectional study	August 2011 to January 2024	3496
141	N’Krumah, 2025	Côte d’Ivoire	Community survey + epidemiological and clinical assessment	multi-year surveillance	795 households
142	Afroz, 2025	Australia	Retrospective registry data analysis	18 years	10,763

### Circumstances of snake bite

Snake bites occurred in a wide range of daily activities, reflecting both occupational and environmental exposure. Agriculture/farming accounted for the largest proportion of incidents (59.1%), followed by working (9.1%) and other works (8.4%). A substantial proportion (5.9%) fell into the “other/unknown” category, indicating either undocumented activity or diverse exposure scenarios (Table 2).

**Table 2:** Circumstances of the snake bites.

Circumstances	Description	N	%
Fishing/ water/swimming in ponds and rivers	The bites occurred primarily while handling snakes caught in fishing nets.	196	0.4
Walking	• Walking barefoot in fields/ hills • Walking on roads and public paths • Walking in the forest • Walking in rural fields during daytime	4039	8.4
Sleeping		1136	2.4
Agriculture/ Farm	• Farming and plantation activities, including field preparation, ploughing, cultivating, weeding, clearing bushes, cutting crops, and harvesting. • Tea, coffee, coconut, rubber, palm, plantation and orchard related work, irrigation tasks, and other field-based manual labour. • Livestock-related activities such as herding and grazing. • Logging, forestry, and other outdoor rural/agricultural work around homes and farmlands.	28315	59.1
Household work	• Cleaning garbage • Indoor domestic chores • Collecting water, firewood for home use • Cooking outdoors • Cutting grass	252	0.5
Handling snake	• Intentional catching/rescuing or killing snakes • Accidental bite while touching/holding/rescuing snake	1737	3.6
Cutting grass/ grazing		367	0.8
Working	• Construction, logging, and other non-agricultural manual labour • Tea factory or plantation (non-field) work • Outdoor occupational work, often without protective footwear • General labour and daily livelihood activities • Non-farm work in forests or other outdoor settings • Timber or saw-mill related activities • Brick-kilns	4303	9.1
Playing	• Playing in gardens/private yards • Playing in forest	153	0.3
Outdoor*		4548	9.5
Other/ Unknown		2825	5.9
Total		**47892**	100.00

*Outdoor activities included exposures in fields, agricultural areas, or during work-related tasks.

Notably, bites during handling snakes (intentional or accidental) contributed to 3.6% of cases, while bites during sleeping/resting accounted for 2.4%. Activities related to fishing or water sources contributed 0.4%, reflecting context-specific livelihood risks. Children were also vulnerable, with 0.3% of bites occurring during play. These findings highlight the importance of behavioural and occupational risk patterns in snake-bite epidemiology.

### Time of snake bite

Of the 15931 cases with time information, day time bites were slightly more (52.6%) than evening bites (47.4%). The highest proportion occurred between 12 PM and 6 PM (39%), followed by 6 PM to 12 AM (29%). Early morning hours (12 AM–6 AM) accounted 12% of bites. This distribution suggests that nocturnal snake activity and human outdoor mobility in evenings significantly contribute to exposure risk. (Table 3)

**Table 3:** Distribution of Snakebite Circumstances and Related Factors.

	N	%
*Time interval where bites have occurred*		
12 AM- 6 AM	923	12
6AM- 12 PM	1561	20
12PM- 6PM	3037	39
6PM- 12 AM	2205	29
Total	7726	100
*Distribution of Snake Bites by Time of Day*		
Day Time	4313	52.6
Evening/Night	3892	47.4
Total	8205	100
*Anatomical Distribution of Snake Bites*		
Upper Limb	16039	19.2
Lower limb	67998	79
Head and neck	458	0.5
Trunk	347	0.4
Unknown/other	751	0.9
Total	85593	100
*Type of Snakes Involved in Snakebite Incidents*		
Sea snakes *sps*.	184	0.16
Cobra family	3316	2.84
Viper/pit viper family.	99382	85.12
Krait family	2182	1.9
Cobra and Krait together	2263	1.9
Other	9432	8.08
Total	116759	100

### Anatomical site of bite

Among 85593 cases with recorded anatomical site, the lower limb was overwhelmingly the most common bite site (79%), followed by the upper limb (19.2%). Bites to the head/neck (0.5%) and trunk (0.40%) were rare. Approximately 0.9% of records listed the site as unknown. The predominance of lower-limb bites aligns with typical exposure patterns during walking, farming, and other ground-level activities. Table 3

### Type of snake involved

From 116,759 records detailing snake type, viper and pit viper species were identified in most incidents (85.12%), followed by cobras (2.84%), kraits (1.9%), and mixed cobra–krait identifications (1.9%). Sea snakes were uncommon (0.16%), while “other” snake types constituted 8.08%. National-level species distribution data showed a heavy predominance of Russell’s viper (51.3%), followed by common krait (22.3%), spectacled cobra (8.9%), and saw-scaled viper (10%). These species correspond to India’s “Big Four,” responsible for most severe envenomation’s and mortality. (Table 3)

Table 4 gives an overview of the snake bite cases from countries like Africa, Australia, Brazil, Costa Rica, France, Lebanon, Mexico, Oman, Nepal, Sri Lanka, USA, Tunisia, Saudi Arabia and Vietnam. The data highlight the global burden of snakebite envenomation and demonstrate considerable variation in epidemiological patterns, healthcare access, species distribution, and clinical outcomes among countries

**Table 4:** Distribution of snake species across different countries.

*Sl No.*	*Country*	*Snake Species*	*n*	*%*	
1.	***AFRICA***	Carpet viper	301	27.17	
		Night Adder	225	20.3	
		Spitting Cobra	171	15.43	
		Cobra	26	2.35	
		Black Mamba	22	1.99	
		Adders	7	0.6	
		Rinkhals	7	0.6	
		Stilleto	3	0.27	
		Viper	96	8.71	
		Forest Cobra	192	17.33	
		Egyptian Cobra	14	1.26	
		Puff Adders	11	0.99	
		Burrowing Adders	30	2.7	
		Egg Eaters	1	0.1	
		Berg Adders	1	0.1	
		Boomslangs	1	0.1	
		**Total**	**1108**		**100**
2.	***AUSTRALIA***	Tiger Snake	944		17.3
		Brown Snake	2350		43.1
		Death Adder	187		3.4
		Sea Snake	61		1.1
		Red Bellied Black Snake	916		17
		Mulga Snake	38		0.69
		Collett’s Snake	6		0.1
		Taipan	141		2.6
		Rough-Scaled Snake	66		1.2
		Hoplocephalus sp.	35		0.6
		Broad Headed Snake	4		0.07
		Stephens-Banded Snake	2		0.04
		Pale-Headed Snake	2		0.04
		Whip Snake	1		0.02
		Eastern Small Eyed Snake	1		0.02
		Non-Venomous	1		0.02
		Unknown sps.	693		12.7
		**Total**	**5448**		**100**
3.	***BRAZIL***	Bothrops	69810		90.4
		Crotalus	952		1.2
		Micrurus	340		0.44
		Brown Banded Water Snake	10		0.01
		Lachesis	5491		7.11
		Philodryas sps.	71		0.09
		Helicops sps.	2		0.002
		Boiruna sps.	1		0.001
		Unknown sps.	510		0.66
		**Total**	**77187**		**100**
4.	***COSTA RICA***	Bothrops	44		97.8
		Lachesis	1		2.2
		**Total**	**45**		**100**
5.	***FRANCE***	*Vipera aspis*	64		
		**Total**	**64**		**100**
6.	***LEBANON***	Viper sps.	1		
		**Total**	**1**		**100**
7.	***MEXICO***	Bothrops	1		
		**Total**	**1**		**100**
8.	***OMAN***	*Echis omanensis*	1		
		**Total**	**1**		**100**
9.	***NEPAL***	Common Krait	71		83.5
		Russell’s Viper	10		11.8
		Cobra	04		4.70
		**Total**	**85**		**100**
10.	***SRI LANKA***	Common Krait	236		44.1
		Hump Nosed Pit Viper	181		33.8
		Russell’s Viper	63		11.8
		Cobra	7		1.3
		Saw-Scaled Viper	1		0.2
		Sri Lankan Pit Viper	1		0.2
		Unidentified	46		8.6
		**Total**	**535**		**100**
11.	***USA***	Rattle Snake	276		19.71
		Copper Head	133		9.5
		Pit Viper	2		0.14
		Native Pit Viper	442		31.6
		Coral Snake	3		0.21
		Cotton Mouth	15		1.07
		Texas Coral Snake	14		1
		Massasauga	474		33.85
		Unknown sps.	41		2.92
		**Total**	**1400**		**100**
12.	***TUNISIA***	*Macrovipera lebetina*	78		92.86
		*Vipera latastei*	6		7.14
		**Total**	**84**		**100**
13.	***SAUDI ARABIA***	Arabian Cobra	5		
		**Total**	**5**		**100**
14.	***VIETNAM***	Krait	585		28.3
		Viper	487		23.6
		Other Snakes	990		48
		Unknown Sps.	02		0.1
		**Total**	**2064**		**100**
15.	***INDIA***	Russel Viper	2165		51.3
		Saw scale	420		10
		Spectacled Cobra	375		8.9
		Hump nosed pit viper (HNPV)	316		7.5
		Common Krait	941		22.3
		Total	4217		100

### Snake–human conflict pathways framework (Fig 3)

The Snake–Human Conflict Pathways Framework illustrates how multiple interacting determinants collectively shape the risk of snakebite. Environmental factors such as seasonality, rainfall, temperature, land-use change, and poor housing influence both snake activity and the ecological contexts in which people live and work. These conditions determine where snakes are found and how often they come into proximity with humans. Occupational exposures particularly agricultural labour, plantation work, forestry, and outdoor manual tasks place individuals directly in snake habitats, increasing the chances of incidental encounters.

**Fig 3:**
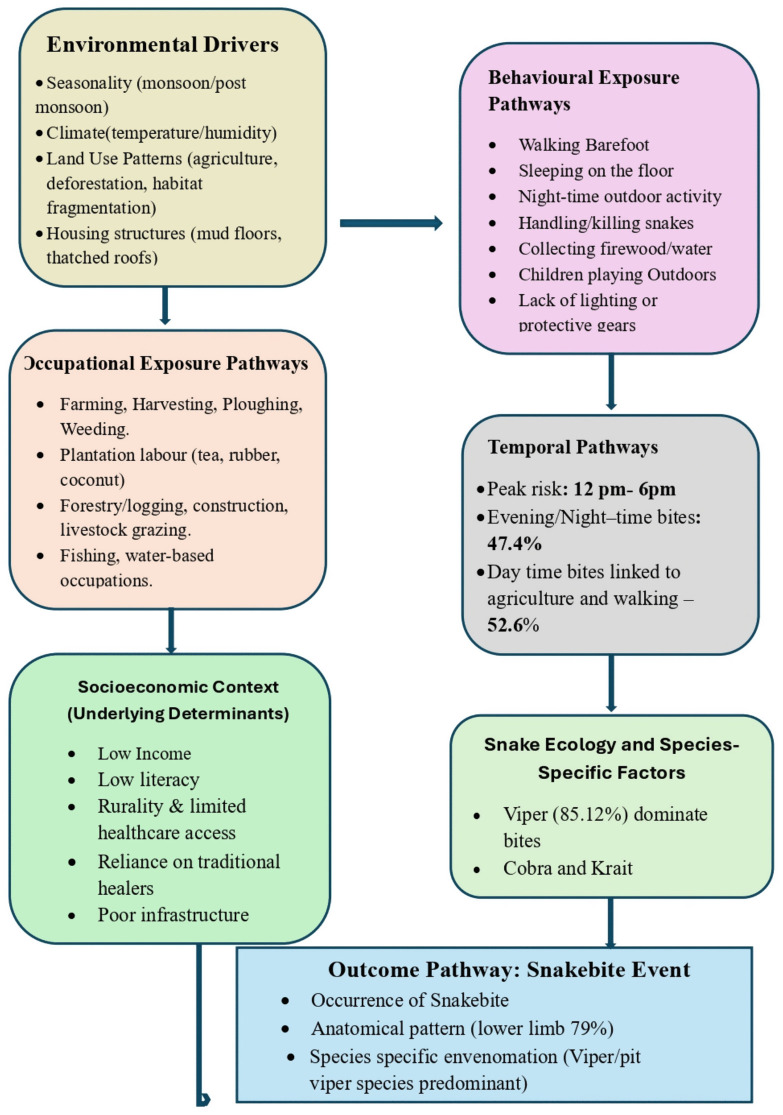
Framework of Snake Human Conflict Pathways Leading to Snakebite Risk.

Behavioural practices, including barefoot walking, night-time outdoor movement, sleeping on the floor, handling snakes, and collecting firewood or water, act as proximal triggers that heighten immediate contact risk. These behaviours are often shaped by broader socioeconomic constraints such as poverty, limited education, inadequate lighting, poor housing infrastructure, and unequal access to healthcare. Socioeconomic vulnerability therefore amplifies both exposure to snake habitats and the likelihood of severe outcomes following a bite.

These determinants interact to produce recurrent snake–human encounters that may culminate in snakebite. The framework highlights that snakebite is a complex, multi-factorial event requiring integrated prevention approaches addressing environmental, behavioural, occupational, and socioeconomic dimensions simultaneously.

Table 5 summarises the key determinants of snakebite by grouping them into four major domains: environmental, occupational, behavioural, and socioeconomic. Environmental factors such as seasonality, rainfall, proximity to forests or agricultural fields, and poor housing structures shape the ecological conditions that increase snake presence around human settlements. Occupational exposures—particularly farming, plantation labour, forestry, construction, and fishing—place individuals directly in snake habitats, making these groups highly vulnerable. Behavioural practices, including barefoot walking, night-time outdoor activity, sleeping on the floor, and handling snakes, act as immediate triggers that heighten the likelihood of snake–human encounters. Socioeconomic determinants such as poverty, limited education, inadequate lighting, poor housing, and restricted access to healthcare further amplify both exposure risk and negative outcomes

**Table 5:** Risk Factors of the Snake Domain.

Risk Factor Domain	Specific Risk Factors	Description of Exposure Context	Supporting Evidence
ENVIRONMENTAL	Seasonality (monsoon, post-monsoon)	Increased snake activity and human agricultural exposure during rainy seasons; flooding displaces snakes into human habitats.	Multiple included studies; epidemiological patterns
	Climatic conditions (temperature, humidity)	Hot and humid climates favour snake movement and human outdoor activity.	Multi-country observational studies
	Proximity to forests, fields, plantations	Higher exposure for communities near agricultural and forest ecosystems.	Consistent across Asian, African, and American studies
	Water bodies (rivers, ponds, irrigation canals)	Increased risk during fishing, washing, or irrigation work.	Circumstances dataset; study characteristics
	Housing structure (mud floors, thatched roofs, open ventilation)	Allows snakes to enter sleeping areas; associated with indoor bites.	Case–control evidence from Bangladesh, India, Sri Lanka
	Land-use change (deforestation, habitat destruction)	Forces snakes into closer proximity with humans.	Ecological and descriptive studies
	Poor lighting in rural areas	Increases night-time encounters with snakes.	Supported by night-time bite distribution
OCCUPATIONAL	Agricultural work (ploughing, weeding, harvesting)	Most common occupational exposure; often barefoot or without PPE.	High proportion of bites in agricultural workers across studies
	Plantation labour (tea, rubber, coconut)	Dense vegetation increases hiding spots for snakes.	India, Sri Lanka data
	Forestry/logging	Encounters during clearing or collecting wood.	Retrospective cohort data
	Construction and manual labour	Snakes disturbed during site excavation or clearing.	Work-related bite patterns
	Livestock herding/grazing	Exposure in fields and forest margins.	Regional reports (Africa, India)
	Fishing/water-based occupations	Handling nets and working in wet habitats increases risk.	0.4% of bites in circumstances dataset
BEHAVIOURAL	Walking barefoot	Major contributor to lower-limb bites; reduced tactile protection.	Epidemiological data: 79% lower-limb bites
	Sleeping on the floor	Increased exposure to nocturnal snake entry indoors.	South Asian case–control evidence
	Outdoor afternoon/evening-time activity	Highest-risk period for snakebites (39% between 12PM–6 PM).	Time-of-bite dataset
	Handling/killing snakes	Provokes defensive bites during intentional encounters.	3.6% of bites involve handling
	Children playing outdoors	Associated with snakebite injuries during unsupervised play.	0.3% of bites in children
	Lack of torch/lighting while walking	Common in rural settings, increasing night encounters.	Supported by nocturnal bite data
	Collecting firewood/water	Frequent rural necessity increasing human movement in snake habitats.	Repeated across observational studies
SOCIOECONOMIC	Low income	Increased engagement in high-exposure occupations; poorer housing.	Bangladesh, India socio-epidemiological studies
	Low literacy/ education	Limited knowledge of snake ecology and prevention.	Mixed-methods and survey studies
	Rurality and remoteness	Limited access to healthcare; delays in antivenom treatment.	Multi-country evidence
	Reliance on traditional healers	Delayed access to hospital care increases morbidity and mortality.	India and Africa reports
	Poor infrastructure (roads, transport)	Slows emergency healthcare access.	Health system analyses
	Inadequate healthcare access	Direct determinant of snakebite outcomes and late presentations.	National surveillance datasets
	Housing quality (low-cost materials, poor maintenance)	Facilitates snake entry into homes and sleeping areas.	Risk identified in multiple cross-sectional studies

## Discussion

This scoping review mapped the breadth and characteristics of evidence on behavioural and environmental circumstances associated with snake–human conflict across diverse geographic and socioecological contexts. Drawing on 142 studies spanning several decades and multiple regions, the synthesis demonstrates that snakebite risk is shaped by a complex interaction of environmental conditions, occupational exposures, behavioural practices, and socioeconomic vulnerability. Rather than occurring randomly, snakebite emerges as a predictable outcome of repeated interactions between humans and snakes within specific ecological and social systems.

Environmental factors were consistently identified as foundational drivers of snake–human conflict. Seasonal patterns, particularly monsoon and post-monsoon periods, were strongly associated with increased snakebite incidence across South Asia, Southeast Asia, Africa, and Latin America.[22] Rainfall and flooding simultaneously increase snake activity and intensify human agricultural and outdoor labour, thereby elevating opportunities for contact. These findings are consistent with ecological studies demonstrating that snake movement and prey availability peak during periods of increased humidity and temperature.[23]

Land-use change, deforestation, and agricultural expansion further intensified snake–human interactions by reducing natural habitats and forcing snakes into closer proximity with human settlements. Housing characteristics—such as mud floors, thatched roofs, open ventilation, and poor structural maintenance—were repeatedly identified as enabling snake entry into domestic spaces, particularly during nocturnal hours. The substantial proportion of bites occurring during sleeping in several South Asian studies reinforces the importance of housing quality as a modifiable environmental determinant [8,24]. However, environmental risk patterns varied across regions, climates, and snake species, highlighting the need for context-specific prevention strategies rather than uniform approaches.

Occupational circumstances represented one of the most consistent and prominent risk domains identified in this review. Agricultural and plantation work accounted for a large proportion of snakebite incidents, reflecting the close overlap between human livelihoods and snake habitats.[18] Activities such as ploughing, weeding, harvesting, and irrigation routinely disturb vegetation and soil, increasing the likelihood of accidental encounters. Similar patterns were observed among forestry workers, loggers, construction labourers, and livestock herders, particularly in forest margins and peri-agricultural landscapes.[11,22]

The predominance of lower-limb bites (over 79% of reported cases) strongly supports an occupational exposure pathway, particularly among individuals working barefoot or with inadequate protective footwear. This anatomical distribution has been consistently reported in previous epidemiological studies and reflects ground-level exposure during walking and field work [18,25]. Importantly, occupational risk extended beyond formal employment to include subsistence activities such as grazing, collecting firewood, and fishing. Together, these findings highlight the need for occupationally tailored prevention strategies, including protective footwear, work-practice modification, and seasonal risk advisories.

Behavioural circumstances acted as immediate triggers that converted environmental and occupational exposure into direct snake–human encounters. Agriculture and farm work particularly barefoot walking associated with snakebite in the compiled dataset.[1] This finding aligns closely with the observed predominance of lower-limb bites and reinforces the role of footwear as a simple but underutilised preventive measure. Afternoon and evening outdoor activity, especially between 12 PM and 6 PM, followed by 6 PM to midnight represented the highest-risk temporal window, corresponding with nocturnal snake activity and limited visibility in rural settings.

Sleeping practices also emerged as an important behavioural determinant. Sleeping on the floor, outdoors, or in poorly secured dwellings increased vulnerability to nocturnal snake entry, a pattern well documented in South Asian case–control studies [9]. Intentional handling of snakes—whether during rescue, killing, or accidental contact—accounted for a notable proportion of bites, highlighting the risks associated with human responses to snake presence. Children’s outdoor play and unsupervised movement further contributed to risk, reflecting age-specific behavioural vulnerabilities. Collectively, these findings suggest that many snakebite events are preventable through targeted behaviour change interventions, including footwear use, improved lighting, safe sleeping practices, and community education on snake avoidance and safe response.

Socioeconomic conditions underpinned and amplified environmental, occupational, and behavioural risk pathways. Poverty constrained housing quality, limited access to protective equipment, and increased reliance on high-exposure livelihoods such as agriculture and manual labour. Low literacy and limited health education reduced awareness of snake ecology, first-aid practices, and appropriate treatment-seeking behaviours. Rurality and remoteness compounded these challenges by delaying access to health facilities and antivenom, particularly in emergency situations.[3,4,24]

The review also highlighted persistent reliance on traditional healers in many endemic regions, often resulting in delayed or inappropriate treatment. These socioeconomic determinants did not merely influence exposure but also shaped outcomes, contributing to higher morbidity and mortality among disadvantaged populations. Importantly, these patterns mirror broader inequities observed in other neglected tropical diseases, reinforcing snakebite as both a biomedical and social justice issue. [1,24]

Temporal analysis revealed a slightly higher proportion of day time bites compared with night time bites, with a clear peak during evening and early night hours. This distribution reflects the intersection of nocturnal snake behaviour and human activities such as evening travel, household chores, and outdoor social or occupational tasks. Species-specific data showed a marked predominance of viper bites, particularly Russell’s viper, followed by kraits and cobras. These species correspond to the “Big Four” snakes responsible for most severe envenoming’s and fatalities in India and parts of South Asia [1,22]. The alignment between species distribution, occupational exposure, and anatomical bite patterns reinforces the ecological plausibility of the findings and underscores the need for region-specific prevention, surveillance, and preparedness strategies. Accordingly, the present findings are not conflicting with the previous study but are complementary, reflecting differences in scope, data aggregation, and the global nature of this analysis. [26]

In Africa, Carpet Viper (27.17%), Night Adder (20.3%), Spitting Cobra (15.43%) and Forest Cobra (17.33%) bites were mainly reported. In Australia, Brown snake constituted 43.1% of bite cases followed by Tiger snakes (17.3%) and Red bellied black snake (17%). Bite cases from Bothrops species were reported in Brazil (90.4%), Costa Rica (97.8%) and Mexico (single case report). Lachesis species constituted, 7.11% and 2.2% of bite cases in Brazil and Costa Rica, respectively. In Nepal (83.5%), Vietnam (28.3%) and Sri Lanka (44.1%), Bite cases by Common Krait was widely reported. Russell’s Viper (11.8%) along with Cobra bite cases (4.7% and 1.3%) were reported in both Nepal and Sri Lanka. In USA, Massasauga (33.85%), Native Pit Viper (31.6%) followed by Rattle Snake (19.71%) bites were reported. While, in Tunisia *Macrovipera Lebetina* (92.86%) bite cases were recorded. A total of 5 Arabian cobra bites were reported in Saudi Arabia and a single bite case of Oman Saw Scaled Viper was reported from Oman. Hump Nosed Pit Viper which is endemic to Western Ghats of India and Sri Lanka contributed to 33.8% of snake bites reported from Sri Lanka (Table 4).

The Snake- Human Conflict Pathways Framework and causal loop diagram developed in this review provide a systems-level interpretation of the findings. They illustrate how environmental change, livelihood patterns, behavioural practices, and socioeconomic vulnerability interact in reinforcing cycles that sustain high snakebite risk. For example, poverty increases engagement in high-risk occupations and poor housing, which elevates exposure and adverse outcomes, further entrenching vulnerability. Such feedback loops highlight why isolated interventions—such as antivenom availability alone—are insufficient to achieve sustained reductions in snakebite burden.

### Implications for policy and practice

The findings of this scoping review have several important implications. First, snakebite prevention must be integrated into broader rural development, agricultural safety, housing improvement, and environmental management programmes. Second, occupational health frameworks should explicitly recognise snakebite as a work-related hazard in endemic regions. Third, community-based education and behaviour-change interventions—tailored to local ecological and cultural contexts—are essential. Finally, strengthening primary healthcare access and emergency referral systems remains critical to mitigating severe outcomes when bites occur.

### Evidence gaps and future research

Despite the extensive body of literature identified, important gaps remain. Many studies lacked standardised definitions of exposure circumstances, limiting comparability across settings. Evidence from parts of Africa and Latin America was less detailed with respect to behavioural context. There is also a paucity of longitudinal and intervention-focused studies evaluating the effectiveness of preventive strategies. Future research should use standardised reporting, mixed methods, and robust evaluation of scalable prevention strategies

### Strengths and limitations

The principal strength of this review lies in its comprehensive scope, systematic methodology, and synthesis of a large and diverse body of literature spanning multiple decades and geographic regions. By mapping behavioural and environmental circumstances across varied contexts, the review provides a consolidated overview of the settings in which snake–human conflict occurs. However, several limitations must be acknowledged. The heterogeneity of study designs, inconsistent reporting of exposure variables, and absence of comparator groups limit causal inference. Geographic imbalance in the evidence base and potential reporting bias further constrain generalisability. As this is a global scoping review, the synthesis is influenced by the predominance of studies from South Asia, particularly India, where reporting largely focuses on the “Big Four” species. Although some non–Big Four species, such as the Hump-nosed pit viper, were noted, an in-depth analysis of other region-specific snake species was undertaken (Table 4). Due to inconsistent country-wise reporting, detailed geographic stratification was not feasible. Consistent with scoping review methodology, no formal critical appraisal of included studies was undertaken. These limitations underscore the need for future research employing standardised exposure definitions, longitudinal designs, and analytical approaches to quantify risk and evaluate prevention strategies.

## Conclusion

This scoping review demonstrates that snake human conflict occurs within consistent environmental, occupational, behavioural, and socioeconomic contexts, providing a structured evidence map to inform prevention rather than causal inference. The findings highlight context-specific opportunities for action, including improved housing and environmental management, safer occupational practices, targeted behaviour change, and strengthened access to timely care. However, the evidence base is disproportionately concentrated in the Indian subcontinent, reflecting the high burden but also limiting global representativeness. To address these gaps particularly in India, which bears a substantial share of global snakebite morbidity and mortality future research should prioritise standardised reporting of exposure circumstances, and integrate ecological and geospatial data to better characterise risk contexts and guide locally relevant prevention strategies.

## Supporting information

S1 File PRISMA checklist. Checklist of PRISMA guidelines for scoping reviews. From: Tricco AC, Lillie E, Zarin W, O’Brien KK, Colquhoun H, Levac D, et al. PRISMA Extension for Scoping Reviews (PRISMAScR): Checklist and Explanation. Ann Intern Med. 2018;169:467–473. https://doi.org/10.7326/M18-0850. This work is licensed under CC BY 4.0.”.
(DOCX)
